# A copy number variant is associated with a spectrum of pigmentation patterns in the rock pigeon (*Columba livia*)

**DOI:** 10.1371/journal.pgen.1008274

**Published:** 2020-05-20

**Authors:** Rebecca Bruders, Hannah Van Hollebeke, Edward J. Osborne, Zev Kronenberg, Emily Maclary, Mark Yandell, Michael D. Shapiro

**Affiliations:** 1 School of Biological Sciences, University of Utah, Salt Lake City, Utah, United States of America; 2 Department of Human Genetics, University of Utah, Salt Lake City, Utah, United States of America; HudsonAlpha Institute for Biotechnology, UNITED STATES

## Abstract

Rock pigeons (*Columba livia*) display an extraordinary array of pigment pattern variation. One such pattern, Almond, is characterized by a variegated patchwork of plumage colors that are distributed in an apparently random manner. Almond is a sex-linked, semi-dominant trait controlled by the classical *Stipper* (*St*) locus. Heterozygous males (Z^*St*^Z^*+*^ sex chromosomes) and hemizygous Almond females (Z^*St*^W) are favored by breeders for their attractive plumage. In contrast, homozygous Almond males (Z^*St*^Z^*St*^) develop severe eye defects and often lack plumage pigmentation, suggesting that higher dosage of the mutant allele is deleterious. To determine the molecular basis of Almond, we compared the genomes of Almond pigeons to non-Almond pigeons and identified a candidate *St* locus on the Z chromosome. We found a copy number variant (CNV) within the differentiated region that captures complete or partial coding sequences of four genes, including the melanosome maturation gene *Mlana*. We did not find fixed coding changes in genes within the CNV, but all genes are misexpressed in regenerating feather bud collar cells of Almond birds. Notably, six other alleles at the *St* locus are associated with depigmentation phenotypes, and all exhibit expansion of the same CNV. Structural variation at *St* is linked to diversity in plumage pigmentation and gene expression, and thus provides a potential mode of rapid phenotypic evolution in pigeons.

## Author summary

The genetic changes responsible for different animal color patterns are poorly understood, due in part to a paucity of research organisms that are both genetically tractable and phenotypically diverse. Domestic pigeons (*Columba livia*) have been artificially selected for many traits, including an enormous variety of color patterns that are variable both within and among different breeds of this single species. We investigated the genetic basis of a sex-linked color pattern in pigeons called Almond that is characterized by a sprinkled pattern of plumage pigmentation. Pigeons with one copy of the Almond allele have desirable color pattern; however, male pigeons with two copies of the Almond mutation have severely depleted pigmentation and congenital eye defects. By comparing the genomes of Almond and non-Almond pigeons, we discovered that Almond pigeons have extra copies of a chromosome region that contains a gene that is critical for the formation of pigment granules. We also found that different numbers of copies of this region are associated with varying degrees of pigment reduction. The Almond phenotype in pigeons bears a remarkable resemblance to Merle coat color mutants in dogs, and our new results from pigeons suggest that similar genetic mechanisms underlie these traits in both species. Our work highlights the role of gene copy number variation as a potential driver of rapid phenotypic evolution.

## Introduction

In natural populations of animals, pigment colors and patterns impact mate choice, signaling, mimicry, crypsis, and distraction of predators [1, 2]. Despite longstanding interest in the spectacular variation in color and pattern among animals, most of we what we know about the molecular mechanisms that mediate vertebrate color patterns comes from a relatively small number of species [3–6]. Understanding the genetic basis of the stunning array of animal color patterns benefits from the study of genetically tractable species; however, progress is hampered, in part, by a limited number of traditional model organisms that show limited variation in color and color patterning. In domestic animals, pigmentation traits are often selected by humans based on colors and patterns they find most attractive. Therefore, domestic species can provide a wealth of information on genetics of color patterning, especially in species with diverse phenotypes among breeds or strains strains [7–9].

The domestic rock pigeon (*Columba livia*) is a striking example of variation shaped by artificial selection, with a multitude of colors and color patterns within and among more than 350 breeds. Because breeds of domestic pigeon belong to the same species and are interfertile, pigeons offer an exceptional opportunity to understand the genetic basis of pigmentation traits using laboratory crosses and genomic association studies [10]. Previously, we identified several genes involved in determining the type and intensity of plumage melanins in pigeons [11, 12], but considerably less is known about the molecular determinants of pattern deposition [13]. The molecular basis of pattern variation is an exciting frontier in pigmentation genetics, and recent work in other vertebrates reveals several genes that contribute to this process; still, the genetic basis of pigment pattern is decidedly less well understood than the genes controlling pigment types [14–23].

**Fig 1:**
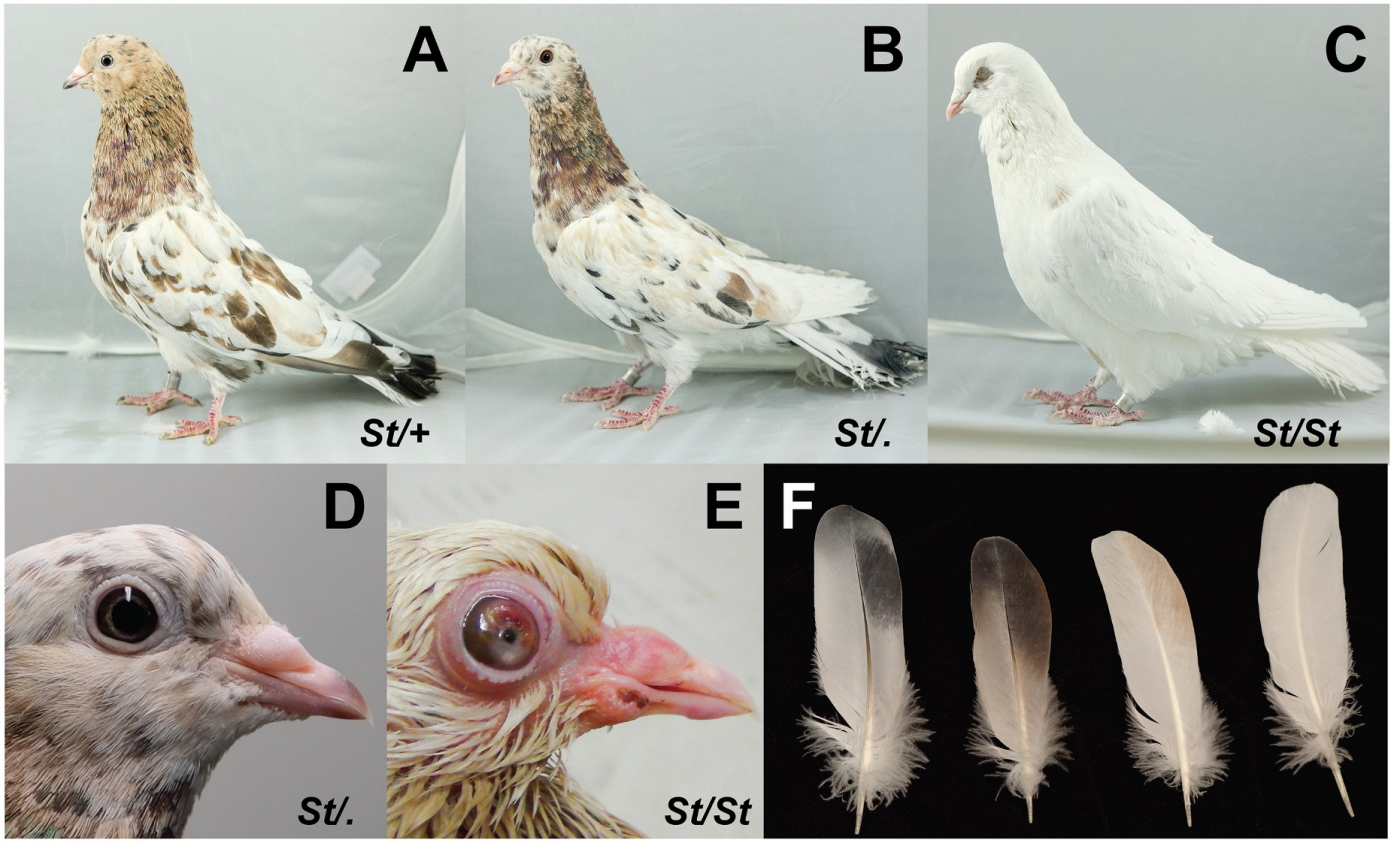
Phenotypes of pigeons carrying Almond alleles (*St*, Almond allele; +, wild type allele). (A) Heterozygous Almond male. (B) Hemizygous Almond female. (C) Homozygous Almond male. (D) Almond females have no observable eye defects. (E) Homozygous Almond males often show severe eye defects. Defects pictured in this juvenile include bloated eyelid and anterior opacity. (F) Wing feathers from different phenotypes, left to right: non-Almond, dark Almond, light Almond, homozygous Almond.

The classical pigmentation pattern in *C*. *livia* known as Almond is caused by a semi-dominant mutation (*St* allele) at the sex-linked *Stipper* (*St*) locus [24] (Fig 1). Unlike most other pigmentation pattern traits in pigeons, the variegated or sprinkled patchwork of plumage colors in Almond is apparently random within and among individuals [25]. Furthermore, the color pattern changes in an unpredictable manner with each molt [26–28]. The number of pigmented feathers in Almond pigeons also increases with each successive molt, and this effect is more pronounced in males [29, 30]. Notably, this phenomenon is the opposite of what is typically observed with pigmentation traits that change throughout the lifespan of an individual, such as vitiligo and graying, which result in a decrease in pigment over time [31–35]. In addition to Almond, at least six other alleles at *St* lead to varying degrees of depigmentation in pigeons, suggesting that the *St* locus might be a mutational hotspot [28, 36].

Heterozygous Almond males (Z^*St*^Z^*+*^) and hemizygous Almond females (Z^*St*^W; males are the homogametic sex in birds), each of which have one copy of the *St* allele, are valued by breeders for their attractive color patterns. However, homozygous Almond males (Z^*St*^Z^*St*^) almost always lack pigmentation in the first set of pennaceous feathers and have severe congenital eye defects [26, 37, 38] (Fig 1B and 1C). The pattern of inheritance of Almond suggests that dosage of the mutant allele, rather than absence of the wild type allele, is responsible for the pigment and eye phenotypes in homozygous males. Eye defects are also associated with pigmentation traits in other vertebrate species, including dogs and horses. The molecular basis of these linked effects are not always known, but some are attributable to melanosome dysfunction [16, 39–44]. Almond pigeons can further illuminate links between pigmentation and eye defects, including whether pleiotropic effects of a single gene or linked genes with separate effects control these correlated traits.

In this study, we investigate the genomic identity of the *St* locus in domestic pigeons. Whole-genome sequence comparisons of Almond and non-Almond birds reveal a copy number variant (CNV) in Almond birds that includes the complete coding sequences of two genes, and partial coding sequences of two others. One of the complete genes, *Mlana*, plays a key role in the development of the melanosome (the organelle in which pigment granules are produced), making it a strong candidate for the pigmentation phenotype observed in Almond pigeons. We also find that different alleles at *St* are correlated with different degrees of expansion of the same CNV, thereby linking a spectrum of pigmentation variants to changes at one locus.

## Results

### A sex-linked genomic region is associated with Almond pigmentation pattern

**Fig 2:**
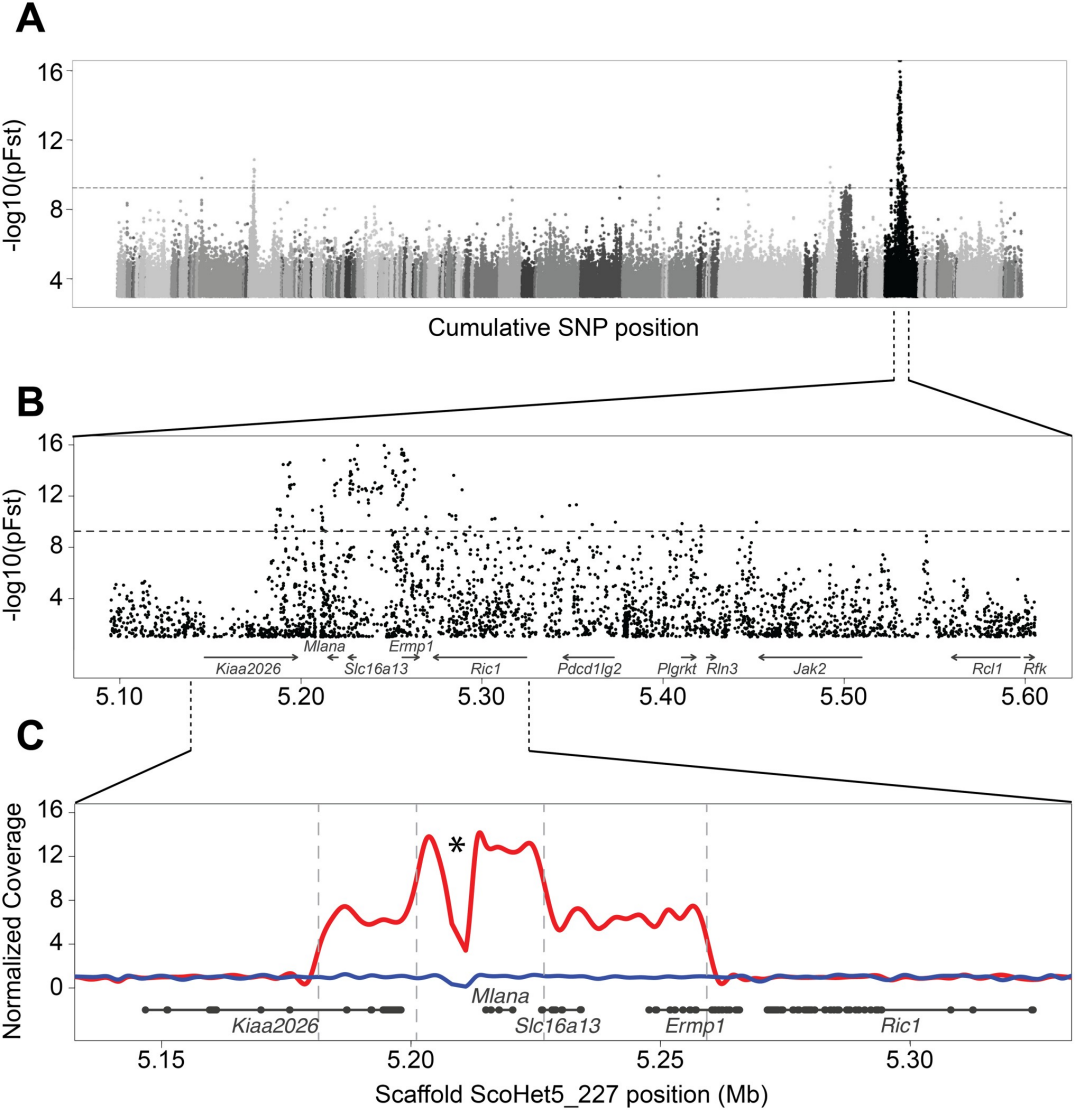
Almond is associated with a CNV on a sex-linked genomic scaffold. (A) Whole-genome pFst comparisons between Almond and non-Almond pigeons. Each dot represents a SNP position, with shades of gray indicating different genomic scaffolds. The horizontal dashed grey line indicates genome-wide significance threshold. -log10(pFst) values lower than 3 are not plotted. (B) Detail of pFst plot for candidate region on ScoHet5_227, a sex-linked scaffold. Gene models are depicted at the bottom of the plot. -log10(pFst) values lower than 1 are not plotted. (C) Detail view of the CNV region. Solid red line represents the mean normalized read depth for 10 female Almond birds in this region. The blue line is a single representative of non-Almond female coverage. Vertical dashed lines indicate positions of CNV breakpoints. Gene models are depicted below the coverage plot in grey (thick lines, exons; thin lines, introns). Asterisk indictates a drop in coverage due to a gap in the genome that was revealed to contain a CR-1 transposable element.

To determine the genomic location of the sex-linked *St* locus, we compared the genomes of 12 Almond pigeons to a panel of 109 non-Almond pigeons from a diverse set of breeds, using a probabilistic measure of allele frequency differentiation (pFst) [45] (see S1 Table for sample details). This whole-genome scan identified several significantly differentiated regions, but one exceeded the others by several orders of magnitude and was located on a Z-chromosome scaffold (ScoHet5_227), as predicted from classical genetics studies (Fig 2A). The differentiated region of ScoHet5_227 (position 5,186,219–5,545,482; peak SNP, *p* = 1.1 e-16, genome wide significance threshold p = 5.5 e-10) contained eight annotated protein-coding genes, none of which had fixed coding changes in Almond compared to non-Almond genomes (VAAST [46]). Therefore, the Almond pigmentation pattern probably does not result from non-synonymous changes to protein-coding genes.

The candidate region included a ~2500-bp gap in the Cliv_2.1 genome assembly, located 3’ of the gene *Mlana* (Fig 2C, asterisk). We amplified and sequenced across this region using PCR and Sanger sequencing, and found that the gap contained a transposable element with a best BLAT [47] match to a CR-1 LINE element of the chicken. PCR amplicons of this region were identical in size (2401 bp) and nearly so in sequence identity (96%) in non-Almond and Almond pigeons. We mapped whole-genome resequencing data from Almond and non-Almond pigeons to this region and found a massive pileup of short-read sequences, suggesting that CR-1-like sequences are abundant throughout the pigeon genome (S1 Fig). These sequences were more abundant in females, perhaps indicating a proliferation of this transposable element on the W-chromosome. Because similar CR-1 elements are present in the candidate region of both non-Almond and Almond genomes, we infer that these elements probably do not contribute to the Almond phenotype. However, we cannot rule out the possibility that subtle differences in sequence have profound effects, or that undetectable changes in a subset of copies of the element in Almond birds (see below) could cause the phenotype.

Several other scaffolds contained sequences that were significantly differentiated between Almond and non-Almond pigeons (Fig 2A). All of these regions are autosomal, and we speculate that they are linked to other color traits that are often co-selected with Almond to give the most desirable sprinkled patchwork of colors, including T-check (a highly melanistic wing pattern [13]; see S2 Fig), kite bronze (a deep reddening of the feathers), and recessive red (a pheomelanic color trait) [25, 28, 36]. However, because Almond is a sex-linked trait [24], we focused our attention on the Z-linked scaffold ScoHet5_227 for further analyses.

### A copy number variant is associated with the Almond pigment pattern

**Fig 3:**
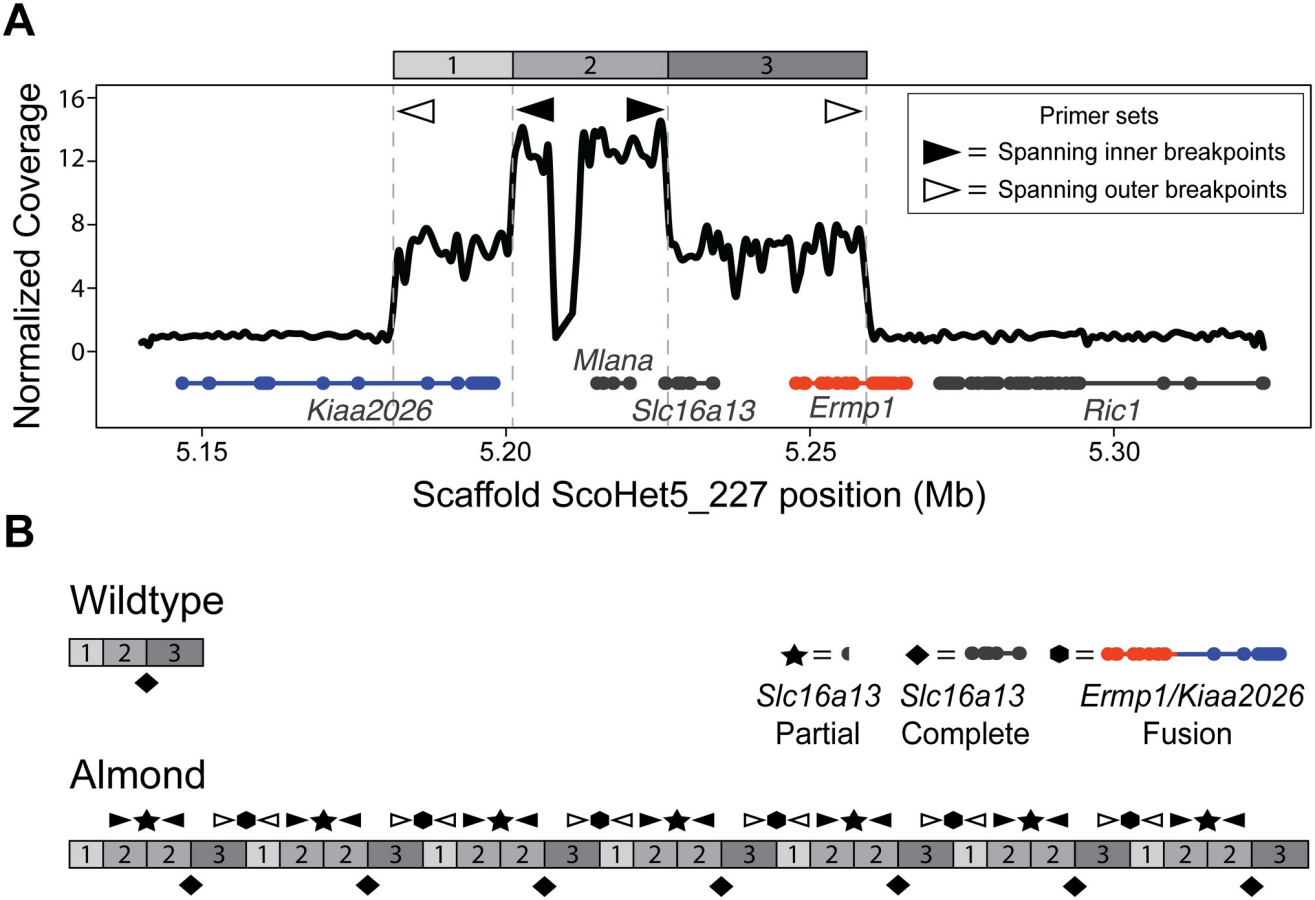
The Almond-associated CNV has a complex structure that results in duplicated, truncated, and fused genes. (A) Coverage diagram showing different regions of the CNV normalized to a non-CNV region on the same scaffold. Two outer regions (1 and 3, above plot) have an approximately 7-fold coverage increase, while one inner region (2) has an approximately 14-fold coverage increase. Gene models are depicted below the coverage plot in grey, orange and blue (thick lines, exons; thin lines, introns). (B) Schematic of the non-Almond (top) and inferred Almond (bottom) structures of the CNV. Gene structural changes resulting from the Almond CNV include a fusion of *Ermp1* and *Kiaa2026* at the segment 3/1 junction (hexagon), and a truncated version of *Slc16a13* at the segment 2/2 junction (star). A complete copy of *Slc16a13* occurs at each 2/3 junction (diamond).

In the absence of fixed coding changes between Almond and non-Almond birds, we next asked if birds with different phenotypes had genomic structural differences in the candidate region. We examined sequencing coverage on ScoHet5_227 and found that all 12 Almond genomes had substantially higher coverage in the Almond candidate region relative to non-Almond genomes, indicating the presence of a copy number variant (CNV) (Fig 2B and 2C). The CNV captures a 77-kb segment of the reference genome (ScoHet5_227: 5,181,467–5,259,256), with an additional increase in coverage in a nested 25-kb segment (ScoHet5_227: 5,201,091–5,226,635). Read-depth analysis confirmed 7 copies of the outer 77-kb segment and 14 copies of the inner 25-kb segment in the genomes of female (Z^*St*^W) Almond pigeons, which have an *St* locus on only one chromosome. We used PCR to amplify across the outer and inner CNV breakpoints of Almond pigeons and determined that the CNV consists of tandem repeats of the 77-kb and nested 25-kb segments (Fig 3).

**Fig 4:**
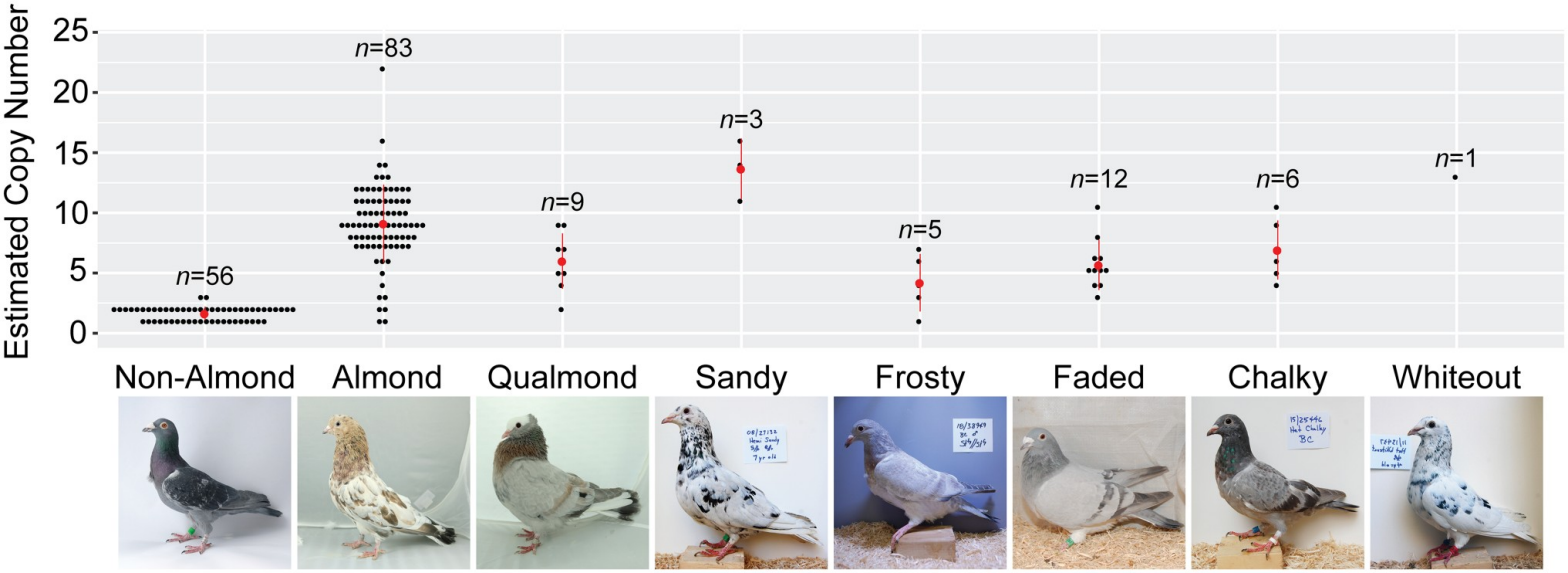
*St*-linked pigmentation phenotypes show quantitative variation in the Almond inner CNV region. Black dots represent results of a TaqMan copy number assay targeting an intron of *Mlana*. Mean copy numbers for each phenotype are shown as red dots. Most individuals without *St*-linked phenotypes have the expected 1 or 2 copies (because *St* is a sex-linked locus, females have a minimum of 1 copy and males have a minimum of 2). All other *St*-linked phenotypes are associated with an expansion of the CNV in the Almond candidate region on scaffold ScoHet5_227, indicating an allelic series at *St*. Numbers above each phenotype indicate number of individuals sampled.

We then genotyped the inner CNV region in a larger sample of Almond pigeons and found a significant association between copy number and the Almond phenotype (TaqMan assay; pairwise Wilcoxon test, *p* = 9.3 e-12). Almost all Almond birds have a copy number that does not overlap with that of non-Almond birds (n = 78 of 83) (Fig 4, S3 Fig; other depigmentation phenotypes are also liked to *St*, as described below). All Almond birds with one or more extra copies of the CNV had both the inner and outer breakpoints (See S2 Table). Nearly all non-Almond birds had only one copy per Z-chromosome (n = 55 of 57). The two non-Almond birds with >1 copy per chromosome had a maximum of one additional copy of the CNV, indicating that small increases in copy number do not necessarily cause the Almond phenotype. These two non-Almond birds have the only the inner portion of the CNV, as determined by PCR assays. In our whole-genome resequencing data, several additional non-Almond birds had small increases in copy number, including two with both the inner and outer breakpoints (S4 Fig, see S2 Table).

Finally, we reasoned that different copies of the CNV could contain different alleles of protein-coding genes, and Almond could be caused by a shared variant in one of the copies of the protein-coding genes that were duplicated. We designed a pipeline to search for low-frequency (4% and higher; see Methods) variants in our whole-genome re-sequencing data. However, we did not find any shared variants in Almond genomes that were absent from non-Almond genomes. For Almond birds with copy numbers that overlapped non-Almond birds but for which we did not have associated whole-genome sequences, we also searched for non-synonymous substitutions in the two full-length genes in the candidate region (*Mlana* and *Slc16a13*), and did not find any changes that were unique to Almond genomes. Overall, these analyses suggest that the Almond phenotype is associated with expansion of the CNV on ScoHet5_227, but not with changes in protein-coding genes.

### Genes within the CNV are misexpressed in Almond feather buds

We next asked if the CNV was associated with gene expression changes between developing Almond and non-Almond feathers. To address this question, we compared expression of genes in the CNV region among birds with (Z^*St*^Z^*+*^, Z^*St*^W, Z^*St*^Z^*St*^) and without (Z^*+*^Z^*+*^ and Z^*+*^W) Almond alleles. We analyzed Almond feather buds with dark and light pigmentation separately to assess whether expression differed between qualitatively different feather pigmentation types, both of which are present in Z^*St*^Z^*+*^ and Z^*St*^W Almond individuals. The CNV contains the complete coding sequences of two genes, *Mlana* and *Slc16a13*, and partial coding sequences of two additional genes, *Ermp1* and *Kiaa2026* (Fig 3A). *Mlana* is predicted to have up to 14 total copies per Z^*St*^ chromosome based on sequencing coverage in Z^*St*^W Almond birds (Fig 3B). *Mlana* is expressed almost exclusively in melanocytes (melanin-producing cells), and encodes a protein that is critical for melanosome maturation through interactions with the matrix-forming protein Pmel [48–50]. Thus, the combination of the biological role of *Mlana* and its location in the Almond CNV makes *Mlana* a strong candidate gene for the Almond phenotype.

**Fig 5:**
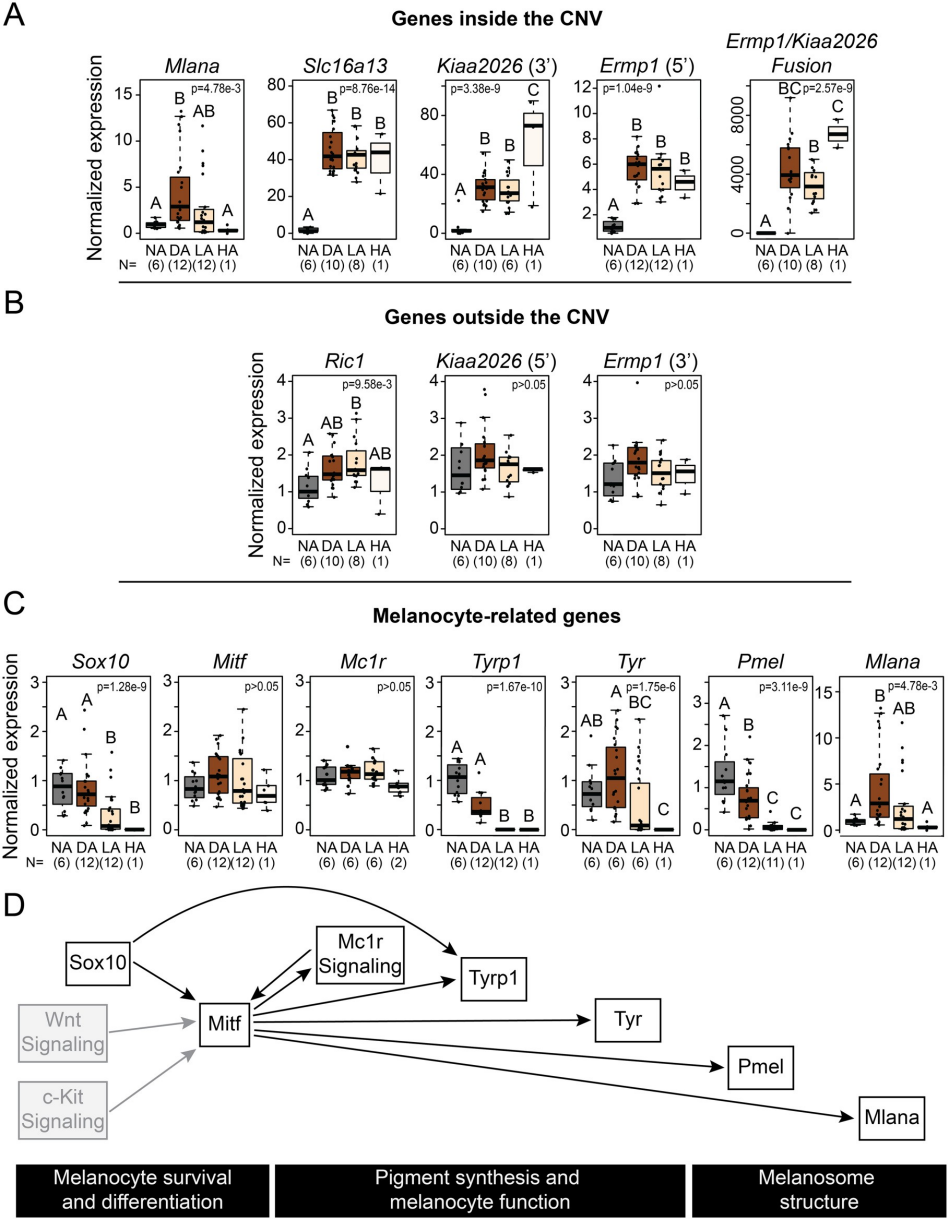
Almond and non-Almond feather buds have distinct gene expression profiles. (A) Exons assayed within the CNV show expression differences in Almond feather buds compared to non-Almond. Boxplots show the results of qRT-PCR assays designed to assess gene expression of exons located in the CNV region. Fusion gene expression results are from qPCR primers spanning exon 7 of *Ermp1* into exon 5 of *Kiaa2026*. (B) Exons assayed outside the CNV show no expression differences in Almond feather buds compared to non-Almond. This indicates expression differences are specific to exons inside the CNV. (C) Expression of melanocyte-related genes. qRT-PCR results indicate a decrease in expression of several genes involved in melanin production in Almond feather buds. (D) Model of interactions among genes and signaling pathways involved in different aspects of pigment synthesis. Gray boxes indicate pathways discussed in the text but not directly represented in our expression analyses. NA, feather buds from non-Almond individuals with wild type alleles at *St*; DA, dark Almond feather buds from hemizygous and heterozygous Almond individuals; LA, light Almond feather buds from hemizygous and heterozygous Almond individuals; HA, feather buds from a homozygous Almond individual. Bar in each box represents the median, box ends indicate upper and lower quartiles, whiskers indicate the highest and lowest value excluding outliers. Different letters indicate groups with statistically significant differences in gene expression determined by ANOVA and post-hoc Tukey test (p<0.05).

Compared to non-Almond feather buds, *Mlana* expression is increased in dark feather buds, but not in light feather buds, from Z^*St*^Z^*+*^ and Z^*St*^W Almond birds or the unpigmented feather buds of homozygous Almond (Z^*St*^Z^*St*^) birds (Fig 5A, S5A Fig; see S3 and S4 and S5 Tables for raw data for all qRT-PCR experiments). (During manuscript revision, another homozygous Almond bird became available and its gene expression profiles are similar to those described here; S5 Fig). We noticed that the variance of expression observed for *Mlana* in both dark and light Almond feather buds, though not statistically significant (Kolmogorov-Smirnov test), trends higher than in non-Almond samples. This data distribution might reflect the variability of the phenotype itself, which is characterized by different quantities and intensities of feather pigmentation both within and between Z^*St*^Z^*+*^ and Z^*St*^W Almond pigeons.

Other genes completely or partially within the CNV show increased expression in feathers from birds with at least one Almond allele relative to non-Almond birds. *Slc16a13* encodes a monocarboxylate transporter, and is predicted to be amplified to six full-length copies in Almond pigeons (Fig 3B). We observed a 40-fold increase in expression of *Slc16a13* in Almond feather buds compared to non-Almond (Fig 5A). *Slc16a13* is not known to be important in pigmentation, and the function of this gene is poorly understood aside from recent GWAS studies implicating its potential involvement in type 2 diabetes [51–56].

In addition to the two genes fully contained within the CNV, a novel fusion of *Ermp1* (a metallopeptidase gene) and *Kiaa2026* (unknown function) is predicted to span the outer CNV breakpoints (Fig 3C). Neither gene is known to play a role in pigmentation or eye development. The predicted Ermp1/Kiaa2026 fusion protein includes a truncated version of Ermp1 with the peptidase domain and 3 of the 6 transmembrane domains (Fig 3B). The 22 amino acids from Kiaa2026 at the C-terminus of the fusion protein are out of normal frame and do not include a known protein domain [57]; thus, the fusion protein is unlikely to create a novel combination of functional domains. As expected, the *Ermp1/Kiaa2026* fusion gene was not expressed in feathers of non-Almond birds, but was expressed in birds with Almond alleles (Fig 5A, S5A Fig). When we analyzed the expression of the exons of *Kiaa2026* and *Ermp1* located outside the CNV, we did not observe expression differences among genotypes (Fig 5B, S5B Fig). Therefore, the Almond CNV is associated with expression of the novel fusion gene, but not with expression differences in the full-length transcripts of either contributing gene. Similarly, *Ric1*, a gene immediately outside the CNV, showed a modest (less than two-fold) expression increase in light Almond feathers relative to other feather types (Fig 5B, S5B Fig). In summary, genes inside CNV showed variable or increased expression in feathers from Almond birds, whereas genes adjacent to the CNV showed little or no expression change.

### Gene expression changes suggest melanocyte dysfunction in Almond feather buds

Plumage pigmentation patterns in Z^*St*^Z^*+*^, Z^*St*^W, and Z^*St*^Z^*St*^ Almond birds are radically different than non-Almond birds, which led us to predict that other components of the melanogenesis pathway might differ as well. The production of melanin by melanocytes is a multi-step process that begins with activation of several pathways, including Wnt and Mc1r signaling, via extracellular ligands and agonists [58–61]. Subsequently, expression of transcription factors, including Mitf, activates a genetic cascade that ultimately promotes the maturation of a functional melanocyte [62]. Within the melanocyte itself, a series of enzymatic reactions and assembly of the melanosome leads to the production and deposition of pigments. Melanosomes are then transferred to skin cells and epidermal appendages, including feathers. In pigeons and other birds with melanin-based pigments, the balance of pheomelanin (reds, yellows) and eumelanin (blacks, browns) deposition determines plumage color [63].

To determine if pigment production signals diverge between regenerating feather buds of Almond and non-Almond birds, we assayed collar cells (a population that contains melanocytes) by qRT-PCR for expression of several marker genes for melanocyte maturation and function. We first examined genes involved in melanocyte survival and differentiation, both of which are critical early events in melanin production. *Sox10*, which encodes a transcription factor that activates expression of many downstream genes including *Mitf*, *Tyrosinase*, and *Tyrp1* expression [64], was downregulated only in light Almond and homozygous Almond feather buds (Fig 5C, S5B Fig). Because *Sox10* regulates *Mitf* and other melanocyte genes, this result indicates that melanocyte dysfunction occurs early in the lightly pigmented Almond feathers, but not in dark Almond feathers. A second melanocyte differentiation and survival marker gene, *Mitf*, encodes a transcription factor that activates expression of *Tyrosinase*, *Tyrp1*, *Pmel*, and *Mlana* [48, 62, 65, 66]. Unlike *Sox10*, *Mitf* was not differentially expressed in any of the phenotypes we tested (Fig 5C, S5C Fig). This result raises the possibility that melanocytes are present in the feathers of all phenotypes, even in severely depigmented feathers [16]. Because *Sox10* was downregulated, we expected *Mitf* to be downregulated as well. However, the persistence of high *Mitf* expression could be the result of activation by other pathways such as Wnt and c-Kit signaling [65]. Another possibility is that high *Mitf* expression from other cell types in the feather collar masks any changes in Almond melanocytes. While *Mitf* is expressed in melanocytes, its expression is not melanocyte-specific [67, 68]. Together, our gene expression results indicate that melanocytes may be present in all feather buds of Almond pigeons (*Mitf* is expressed), but decreased *Sox10* expression in light and homozygous Almond feathers suggests multiple copies of the Almond CNV are associated with dysfunction early in melanogenesis in light and homozygous Almond feathers (*Sox10* expression is decreased).

We next assayed genes involved in pigment production, an indicator of melanocyte function. *Mc1r*, which encodes a G-protein-coupled receptor necessary for eumelanin production [69], was not differentially expressed among phenotypes (Fig 5C, S5C Fig). Like our expression results for *Mitf*, our *Mc1r* results suggest that melanosomes are, at least, present in the feather buds of all genotypes. *Tyrosinase*, which encodes a critical enzyme for both eumelanin and pheomelanin production, was significantly downregulated in homozygous Almond feather buds, and highly variable in light and dark Almond feather buds (Fig 5C, S5C Fig). *Tyrp1*, which encodes another enzyme important for eumelanin but not pheomelanin production [70], was downregulated in all Almond feather buds, with the most severe effects in light and homozygous Almond feather buds (Fig 5C, S5C Fig). Thus, the eumelanin synthesis pathway is affected in all Almond feathers, but pigment generation and melanocyte function genes are more impacted in light Almond and homozygous Almond feather buds, with the most severe gene downregulation observed in homozygotes (Fig 5C, S5C Fig).

Finally, we measured expression of the melanosome structure gene *Pmel*, which encodes an amyloid protein that forms part the melanosome matrix [71–73]. Our candidate gene *Mlana* encodes a protein that interacts with Pmel and is also critical for melanosome matrix formation. We found that *Pmel* was downregulated in all Almond feather buds, and most severely in the two most depigmented types, light Almond and homozygous Almond (Fig 5C, S5C Fig). As described above, *Mlana* expression increased in dark Almond feathers but was similar to non-Almond in light Almond and homozygous Almond feather buds. These results are difficult to reconcile because these two genes are regulated by Mitf. Nevertheless, our results show that even the pigmented feathers in Almond birds show altered expression of pigmentation genes.

In summary, in homozygous Almond feather buds, the pigmentation production pathway is altered at an earlier stage of eumelanogenesis than in heterozygous and hemizygous Almond birds. In birds with one copy of the Almond allele (Z^*St*^Z^*+*^ and Z^*St*^W) light feathers show downregulation of more eumelanin production genes than do dark feathers. Thus, phenotypically different Almond feathers have distinct pigmentation gene expression profiles.

### Other alleles at the *St* locus are copy number variants

Classical genetic studies point to multiple depigmentation alleles at the *St* locus [27, 36, 74, 75]. To determine if the Almond CNV is associated with these other alleles as well, we genotyped pigeons with other *St*-linked phenotypes and found significant increases in copy number of the inner region in Qualmond (*St*^*Q*^; N = 9, *p* = 3.27 e-03) and Faded (*St*^*Fa*^; N = 12, *p* = 8.9 e-04) pigeons compared to those without *St*-linked phenotypes (Fig 4, S4 Fig, S2 Table). Sandy (*St*^*Sa*^; N = 3, *p* = 0.1), Frosty (*St*^*fr*^,N = 5, p = 0.1), Chalky (*St*^*C*^; N = 6, *p* = 0.1), and White Out (N = 1, p = 0.96) showed a trend of copy number increase that did not reach significance. Together, these results demonstrate that copy number increase is associated with a variety of depigmentation alleles at the *St* locus.

We also tested whether different *St* alleles share the same CNV breakpoints. We amplified and sequenced across the Almond CNV breakpoints in Qualmond (N = 4), Sandy (N = 2), Faded (N = 2), and Chalky (N = 4) pigeons and found that the breakpoints are identical in all phenotypes tested. Therefore, a single initial mutational event was probably followed by different degrees of expansion in different *St* alleles. Notably, the breakpoints of the 77-kb segment (ScoHet5_227: 5,181,467 and 5,259,256) are enriched for CT repeats. These repeat sites could facilitate non-allelic homologous recombination, which could have generated the *St* allelic series [76].

## Discussion

### *Mlana* is a strong candidate gene for the Almond phenotype

We identified a CNV associated with plumage pigmentation variation and an eye defect in domestic pigeons. Different numbers of copies of this structural variant are associated with a series of depigmentation alleles at the same locus. In the feathers of Almond birds, the CNV is associated with changes in the expression of genes within its bounds.

One of these genes, *Mlana*, is a strong candidate for Almond due to its role in melanosome maturation. *Mlana* and *Pmel* are co-regulated by Mitf and their protein products physically interact with each other during the process of matrix formation in the melanosome [49, 77]. Expression of *Pmel* and proper formation of the melanosome matrix is crucial for eumelanin deposition, but not for the development of pheomelanic melanosomes [78–80]. As a result, *Pmel* mutations cause eumelanin defects in cattle, chicken, and mouse [81–84]. Feathers of Almond pigeons tend to have a pheomelanic appearance (Fig 1). We postulate that changes in matrix development could contribute to the reduction of eumelanin without a corresponding change in pheomelanin; however, we have not measured eumelanin and pheomelanin content directly.

Melanosome matrix defects can also have pleiotropic effects beyond the skin and its appendages. For example, *PMEL* mutations are linked to pigmentary glaucoma in humans [85]. More strikingly, *Pmel* mutations in horse, dog, and zebrafish result in both epidermal pigmentation phenotypes and eye defects, similar to Almond pigeons [44, 85–90]. The merle coat pattern in dogs is associated with a transposon insertion in an intron of *PMEL*, resulting in a non-functional PMEL protein and a phenotype that is remarkably similar to the Almond phenotype in pigeons [44, 87]. Dogs homozygous for the *PMEL* mutation, much like homozygous Almond pigeons, are severely hypopigmented. Additionally, homozygous *PMEL* mutant dogs have eye defects, such as increased intraocular pressure, ametropia, microphthalmia, and coloboma [91]. The observation that Pmel, which interacts directly with Mlana, is repeatedly connected to both pigmentation and eye defects makes *Mlana* a strong candidate for similar correlated phenotypes in Almond pigeons. Likewise, in humans and mice, mutations in melanosome genes (e.g., *Oca2*, *Slc45a2*, *Slc24a5*) produce both epidermal depigmentation and eye defects, thereby further demonstrating a shared developmental link between these structures [92–94].

The other full-length gene within the CNV, *Slc16a13*, does not have a known role in pigmentation. In humans, SLC16A13 localizes to the golgi apparatus and increases in expression in response to a nutrient nuclear receptor agonist in the small intestine [55]. *Slc16a13* is implicated in genome-wide association studies of type 2 diabetes [53–56, 95], but it is unclear what function this gene might play in pigmentation or eye development. Nevertheless, in regenerating Almond feathers, *Slc16a13* expression increases substantially (40-fold) relative to non-Almond feathers, raising the possibility that this gene is somehow involved in the Almond phenotype. An increase in *Slc16a13* expression could drive components of the Almond phenotype in feathers, eyes, or perhaps both. We also cannot rule out the possibility that *Ermp1*, *Kiaa2026*, or their fusion gene could play a role in the Almond phenotype. However, none of these genes are particularly strong candidates for pigmentation change or eye defects. Furthermore, given the linked pigment and eye phenotypes observed in *Pmel* mutants in other species, *Mlana* alone could be sufficient to induce both pigmentation and eye defects in Almond pigeons. Future work, such as transfecting and overexpressing genes from the CNV in a pigment cell line, will explore the effects of these genes on cell survival and pigmentation.

Nearly all Almond pigeons in our study had a pronounced expansion of the CNV that was associated with the Almond phenotype. However, a small number of the pigeons in our dataset were identified as Almond but had copy numbers that matched non-Almond pigeons. This situation was also observed in a recent pedigree-based study of the *St* locus [96]. A trivial explanation for this discrepancy in our study is that some of the birds were misphenotyped by their breeders, and were not actually Almond. However, more biologically interesting explanations could also be at play, but we are currently underpowered to detect either of them. One possibility is that Almond arises from a heterogenous collection of alleles at the *St* locus, some of which are not caused by the canonical Almond CNV. We already have observed similar situations in pigeons, in which different deletions of a *Sox10* (*E* locus) melanocyte enhancer are associated with the recessive red phenotype, and several alleles of *Tyrp1* (*B* locus) are linked to brown plumage [11]. A second possibility is that a different locus produces an Almond-like phenotype in a small subset of birds. Analogously, a transposon insertion at the *SMOC2* locus is associated with brachycephaly in most–but not all–dogs [97].

### Gene expression is altered in Almond birds

In other organisms, copy number variation can result in gene expression changes in the same direction as the copy number change (i.e., the presence of more copies is correlated with higher expression) [98–100]. We observed a similar trend of higher expression of genes captured in the Almond-linked CNV (Fig 5A, S5A Fig). In contrast to this trend, however, *Mlana* showed an increase in expression in dark Almond feathers, but not in light Almond or homozygous Almond (unpigmented) feathers. *Mlana* is also the gene with the greatest copy number increase, with up to 14 copies in hemizygous Almond genomes and 28 copies in the homozygous Almond genome.

With the above observations of gene expression in mind, why might homozygous Almond birds lack *Mlana* expression in feather buds when they have 28 copies of the gene? One possibility is epigenetic silencing. High copy numbers in tandem arrays induce gene silencing in several organisms [101–105]. In fruit flies, for example, tandem arrays lead to variegated gene expression of the white eye gene [102]. This change in expression, in turn, leads to mosaic eye color, a scenario reminiscent of the color mosaicism in the feathers of Almond pigeons. In mouse, experimentally reducing the number of copies of *lacZ* in a tandem array causes an increase in gene expression, indicating that reducing copy number may relieve gene silencing [104]. Likewise, it is possible that somatic copy number decrease could relieve gene silencing and restore higher expression of *Mlana* in dark Almond feather buds.

Another potential explanation for the lack of *Mlana* expression in homozygous Almond feathers is cell death or immunity-mediated destruction of melanocytes. Overexpression of *Mlana* could have a toxic effect on cells, leading to cell death before melanocyte maturation. Similarly, in humans, overexpression of genes is often associated with disease [106–108], and in yeast, overexpression of genes can reduce growth rate [109]. Alternatively, Almond melanocytes might elicit an autoimmune response, similar to the destruction of melanocytes in human pigmentation disorders. MLANA is a dominant antigenic target for the T cell autoimmune response in human skin affected by vitiligo [110, 111], and perhaps the presentation of Mlana antigens in Almond pigeons elicits a response that depletes melanocytes in the developing feather buds. A potentially analogous autoimmune response depletes the melanocyte population and mimics vitiligo in Smyth line chickens [112]. This hypothesis has the caveat that *Mitf* does not show differential expression among phenotypes, suggesting that melanocyte survival is similar in Almond and non-Almond feathers; however, as discussed above, our *Mitf* expression assays might include transcripts from cells other than melanocytes in the feather bud collar.

If genes in the CNV are being randomly silenced in Almond pigeons, or cells with high expression are escaping cell death in a random manner, then we might expect to see high variance in gene expression among Almond feather samples. Consistent with this prediction, the variance in expression of *Mlana* in both dark and light Almond feather buds trends higher than in non-Almond samples (Fig 5A). This variance might also explain the random pattern of pigmentation and de-pigmentation observed among the feathers of these birds, and even among successive generations of feathers from the same follicle. If each cell population is affected differently due to stochastic events resulting in differential expression, then random pigmentation patterns could be the outcome.

### CNVs as mechanisms for the rapid generation of new phenotypes

In addition to finding a CNV at the *St* locus in Almond birds, we found quantitative variation in copy number among other alleles at this locus. Variation at this CNV may have a quantitative effect on de-pigmentation, with the degree of copy number increase correlating with degree of depigmentation and eye defects. For example, pigeon breeders report that Sandy and Whiteout–two phenotypes with among the highest numbers of copies of the CNV (Fig 4)–have associated eye defects similar to Almond (Tim Kvidera, personal communication) [36, 74]. Although we currently have a small sample size of other *St*-linked phenotypes, we see a trend that other alleles produce milder pigment phenotypes and have less CNV expansion than the Almond allele. Similar quantitative effects of CNVs occur in other organisms as well, including a correlation between comb size and copy number of *Sox5* intron 1 in chickens [113].

While birds with different breeder-identified *St* phenotypes can look very different (e.g., Fig 4), it is also possible that there is no clear genetic distinction between *St* alleles. Instead, it is possible that copy number variation couples with genetic variation at modifier loci to produce different phenotypes. For example, we know that breeders select for T-check wing shield pattern alleles in their Almond lines [25, 28, 36], and we confirmed enrichment of these alleles in our whole-genome resequencing data (S2 Fig). Variation at other loci that control aspects of pigment production or deposition, coupled with expansion or contraction of the CNV, could contribute to the array of phenotypes attributed to the *St* allelic series. In the future, we hope to acquire sufficient sample sizes of other *St*-linked phenotypes to compare selection on modifiers, and confirm the inheritance of phenotypes other than Almond in controlled crosses.

Other evidence underscores a central role for the CNV itself in generating variation. Pigeon breeders have reported that parents with one *St*-linked phenotype can produce offspring of another phenotype in the *St* series [36, 114]. Specifically, Faded, Qualmond, and Hickory (an *St*-linked phenotype not analyzed in this study) pigeons have produced Almond offspring. These classical breeding studies suggest that allelic conversion can occur rapidly and, based on our finding of copy number variation among *St* alleles, may result from simple expansion or contraction of a CNV. In another striking similarity between Merle dogs and Almond pigeons, germline expansions or contractions of Merle alleles of *PMEL* result in a spectrum of coat pattern phenotypes that can differ between parents and offspring [87, 115]. Thus, unstable CNVs like the one we found at the *St* locus may provide a mechanism for extraordinarily rapid phenotypic diversification in pigeons and other organisms [116–119]. Sometimes these changes are favored by natural selection in wild populations, whereas in the case of domestication phenotypes like Almond, favorable changes are accompanied by deleterious pleiotropic effects that rely on human intervention for long-term maintenance.

## Materials and methods

### Ethics statement

Animal husbandry and experimental procedures were performed in accordance with protocols approved by the University of Utah Institutional Animal Care and Use Committee (protocols 10–05007, 13–04012, and 16–03010).

### DNA sample collection and extraction

Blood samples were collected in Utah at local pigeon shows, at the homes of local pigeon breeders, and from pigeons in the Shapiro lab. Photos of each bird were taken upon sample collection for our records and for phenotype verification. Breeders outside of Utah were contacted by email to obtain feather samples. Breeders were sent feather collection packets and instructions, and feather samples were sent back to the University of Utah along with detailed phenotypic information and genetic relatedness. DNA was then extracted from blood, as previously described [11]. DNA from feathers was extracted using the user developed protocol for Purification of total DNA from nails, hair, or feathers using the DNeasy Blood & Tissue Kit (Qiagen Sciences, Germantown, MD).

### Genomic mapping analyses

BAM files from a panel of previously resequenced birds were combined with BAM files derived from new sequences from 11 Almond females and 16 non-Almond birds aligned to the Cliv_2.1 genome assembly [120] (new sequence accessions: SRA SRP176668, accessions SRR8420387-SRR8420407 and SRR9003406-SRR9003411; BAM files created as described previously [13]). SNVs and small indels were called using the Genome Analysis Toolkit (Unified Genotyper and LeftAlign and TrimVariants functions, default settings [121]). Variants were filtered as described previously [45] and the subsequent variant call format (VCF) file was used for downstream analyses.

Whole genomes of 12 Almond and 96 non-Almond birds were tested for allele frequency differentiation using pFst (VCFLIB software library, https://github.com/vcflib; see S1 Table for sample information) [45]. For analysis of fixed coding changes, VAAST 2.0 [46] was used to conduct an association test and to search for putative disease-causing genetic variants common to all Almond individuals but absent from non-Almonds. Annotated variants from affected individuals were merged by simple union into a target file. The background file included variants from 66 non-Almond birds, while the target file contained variants from the 12 Almond birds. VAAST analysis revealed that there were no fixed genetic variants among the Almond individuals that were absent in the background dataset.

### Detection of Almond-specific, low-frequency polymorphisms

To identify low-frequency polymorphisms in almond birds, we used BCFtools [122] mpileup to identify polymorphisms and calculate the allelic depth of each polymorphic site within the CNV region (ScoHet5_227:4821266–5696554) for 10 Almond and 14 non-Almond birds. We included any allele that represented more than 4% of the total read depth for any individual at that site (bcftools filter -i "AD[1]/DP > 0.04"). This allowed us to keep alleles that would be present in 1 out of 14 copies (~7%; maximum number of copies of the inner CNV was 14 in the hemizygous females assayed) while removing sequencing errors. We examined the output file and split it into variant sites with only two alleles and sites with more than two alleles.

For variants with only 2 alleles, we used the VCFtools “--freq” option to calculate allele frequency separately in Almond and non-Almond populations, and then compared allele frequecnies between the two populations. We did not identify any variants that were specific to Almond birds. For variants with more than 2 alleles, we assessed the allelic depth for each individual allele in Almond and non-Almond populations. We used a custom R script to extract allelic depth from the VCF file for each Almond individual. For each site in each individual, we coded alleles as 1 for “present,” where at least 1 read contained the allele, or 0 for “absent,” where no reads contained the allele. We then summed these scores across all 10 Almond individuals to identify low-coverage alleles that were present in three or more Almond birds. We next assessed the presence of each of these low-coverage alleles that appear in three or more Almond birds in the non-Almond samples. We found that all low-frequency alleles present in three or more Almond birds are also present in non-Almond birds at variable coverage and frequency. Therefore, we did not find any evidence that Almond pigeons harbor any low-frequency, Almond-specific variants in the CNV region.

To test for coding mutations that could explain the Almond phenotype in Almond birds with copy numbers that overlap with non-Almond birds (1–3 copies of the *Mlana* region), we examined the predicted coding sequences of *Mlana* and *Slc16a13*, the two complete genes in the CNV. We assayed an Almond female with one copy, an Almond male with 2 copies, an Almond male with 3 copies, and an Almond female with 3 copies, but did not find any Almond-specific substitutions, Sequences for *Mlana* (accession numbers MN862483-MN862487) and *Slc16a13* (MN862488-MN862492) were deposited in Genbank.

### Sequencing across the genomic gap in the Almond candidate region

To determine the content in the gap region of the genome within the Almond CNV, we designed primers specific to the sequence flanking the gap region to amplify the entire region (S6 Table). Using Sanger sequencing, we sequenced into the gap region from both sides in a non-Almond bird, then used these results to design additional primers as we walked across the gap. We used Sequencher v5.4.1 [123] to align and assemble all Sanger reads and found that the sequence within the gap region was 2401 bp in length (Genbank accession numbers MN862493 and MN862494). This result was similar to the expected length of the gap based on the number of “N” placeholder basepairs in the genome assembly (2496 bp). We used BLAT [47] to compare our 2401-bp gap region sequence to the chicken genome and found that it contains a CR-1 like a transposable element. We then generated a 245-kb reference genome for the almond CNV and flanking region that includes the previously missing gap sequence, and used Bowtie2 to align raw Illumina sequence reads from 14 Almond and 4 non-Almond birds to this region. We analyzed read depth using Samtools and found a 500x increase in coverage in the gap region in both non-Almond and Almond birds, indicating this region is likely similar to other regions in the genome containing CR-1 like elements (this common, repetitive element probably explains some of the difficulty in resolving this genomic region based on the short reads used to assemble the reference genome). We then used the primers designed for Sanger sequencing of the gap region to sequence this region in an Almond bird.

### CNV breakpoint identification and read-depth analysis

Read depth in the CNV-containing region was analyzed in 12 Almond and 118 non-Almond resequenced whole genomes. Scaffold ScoHet5_227 gdepth files were generated using VCFtools [124]. Read depth was normalized using a region (scaffold ScoHet5_227: 1–5,000,000) that did not show an increase in sequencing coverage in Almond genomes.

To determine the CNV breakpoints, we first identified the region of increased sequencing coverage in Almond genomes using the depth function in VCFtools [124]. Next, we examined BAM files of Almond genomes in IGV [125] in the region of coverage increase, and identified locations at which reads were consistently split (did not map contiguously). These locations were the putative breakpoints. We then designed PCR primers that amplify 1-kb products spanning the putative breakpoints (see S6 Table for primer sequences). Finally, we used PCR to amplify across the putative breakpoints. PCR products were purified and sequenced, and aligned to the pigeon genome assembly using Blast+ version 2.7.1 [126]. The CNV breakpoint primers (see Fig 3B) successfully amplified products in 40 of 43 Almond pigeons tested.

### Fusion gene analysis

The putative mRNA sequence of the *Ermp1/Kiaa2026* fusion gene was determined by concatenating the mRNA sequence of the exons on one side of the outer breakpoint with the exons that map to the outer breakpoint. The fusion of these exons was confirmed using exon spanning primers and qPCR (See S6 Table for primer sequences). The putative mRNA sequence was translated, and then analyzed for domains using HMMER searches in SMART (Simple Modular Architecture Research Tool) [57]. We searched for domains in the SMART database, and also searched for outlier homologs, PFAM domains, signal peptides, and internal repeats.

### Taqman assay for copy number estimates

Copy number variation was estimated using a custom Taqman Copy Number Assay targeted to the *Mlana* region (MLANA_CCWR201) for 150 Almond, 9 Qualmond, 3 Sandy, 14 Faded, and 6 Chalky, 5 Frosty, and 56 individuals without *St*-linked phenotypes. Following DNA extraction, samples were diluted to 5 ng/uL and run in quadruplicate according to manufacturer’s protocol. Copy number was determined using CopyCaller Software v2.1 (ThermoFisher Scientific, Waltham, MA). An intron in *RNaseP* was used for normalization of copy number. Sigificant increase in copy number was determined by a pairwise Wilcoxon test with Bonferroni correction.

### RNA isolation and cDNA synthesis

To assay gene expression, secondary covert wing feathers were plucked to stimulate regeneration and allowed to regenerate for 9 days (see S3 Table for sample details). Nine-day regenerating feather buds were plucked, then the proximal 5 mm was cut and stored in RNA later at 4°C overnight. Feather buds were then dissected and collar cells removed, and stored at -80°C until RNA isolation. RNA was then isolated and reverse transcribed to cDNA as described previously [11].

### qRT-PCR analysis

cDNA was amplified using intron-spanning primers for the appropriate targets using a CFX96 qPCR instrument and iTaq Universal Sybr Green Supermix (Bio-Rad, Hercules, CA) (S6 Table). Samples were run in duplicate and normalized to β-actin (see S4 and S5 Tables for raw results). Results were compared in R [127] using ANOVA, followed by a Tukey post hoc test to determine differences between phenotypic groups. Differences were considered statistically significant if *p* < 0.05. Primers used for each gene are included in S6 Table.

qPCR analysis for a second homozygous Almond bird was completed after the initial experiments. Because this was an independent experiment and could not be combined with previous qPCR results, we ran the second homozygous Almond pigeon feather bud cDNA (n = 2 feather buds) alongside feather bud cDNA from a non-Almond pigeon (n = 2 feather buds), dark feathers from a Almond pigeon, and the original homozygous Almond (n = 2 feather buds), and ran a t-test to determine if the two homozygous Almond birds were statistically distinguishable.

## Supporting information

S1 Table NCBI SRA submission numbers and breed information for birds used for genomic analysis in this study. (XLSX)

S2 Table Copy number results from Taqman assay of *Mlana* region. (XLSX)

S3 Table Sample sizes and identifiers of birds included in each phenotypic category for qRT-PCR analysis in Fig 5. (XLSX)

S4 Table Raw qRT-PCR results for Fig 5. (XLSX)

S5 Table Raw qRT-PCR results for S5 Fig. (XLSX)

S6 Table Primer sequences used in this study. (XLSX)

S1 Fig Depth plots of whole-genome sequencing data around genomic gap region in the Almond CNV. This region was found to contain a CR-1-like element. Plots show normalized read-depth for resequenced pigeon genomes titled by SRA accession number. Reads were aligned to the Almond CNV region, with the gap in the genome assembly (Cliv_2.1) bridged with sequence obtained from Sanger sequencing. Representative non-Almond individuals are shown in the left column, with Almond individuals on the right. The top two rows are male, and the bottom two rows are females. The x-axis is the distance from the gap region sequence, and the y-axis shows coverage depth, normalized to the first 10000 bp of the region. All individuals have a spike in coverage in the region containing the CR-1-like transposable element sequence, with females showing a greater increase than males. The greater increase in females could be due to an abundance of CR-1-like transposable element sequence on the W chromosome.
(PDF)

S2 Fig Almond is associated with T-check (and/or checker) pattern alleles at the *C* locus on scaffold ScoHet_527 (red box). We repeated the association test shown in Fig 2A, except T-check and checker birds were removed from the background (non-Almond) population. We chose this example to show selection on a modifier of Almond because we previously identified the molecular basis for this trait and knew its genomic location. The Almond-associated peak remains in the same location toward the right side of the plot. Different shades of gray indicate different genomic scaffolds (same order as Fig 2A), and the horizontal dashed grey line indicates the genome-wide significance threshold.
(PDF)

S3 Fig *St*-linked pigmentation phenotypes show quantitative variation in the Almond CNV. Black dots represent results of a TaqMan copy number assay. Mean copy numbers for each phenotype are shown as red dots. These are the same data shown in Fig 4 separated by sex.
(PDF)

S4 Fig Low levels of copy number increase of the Almond-associated CNV is found at low frequency in non-Almond pigeons. Plots in each panel compare coverage of a representative non-Almond individual (red) to mean normalized read depth for 10 female Almond birds (black) in the Almond CNV region of ScoHet5_227. SRA accession numbers for non-Almond birds are indicated above each panel. Grey dashed line at y = 1 is the coverage level expected if there is no expansion of the CNV. (A) Coverage plot for a non-Almond bird with a coverage increase in just the inner CNV region. Similar coverage increases were found in 2 out of 131 individuals analyzed in the whole genome resequencing panel (SRS346902, SRS2803087). (B) Coverage plot for a pigeon that has coverage increase of the entire CNV region but does not have the inner nested CNV duplication within the larger CNV. This configuration was found in 5 individuals in the whole genome resequencing panel (SRS346897, SRS346875, SRR8430387, SRS346889, SRS2803080). (C) Coverage plot for a non-Almond bird that has expansion of the outer CNV and further duplication of the inner CNV region. This configuration was found in two individuals from our whole genome resequencing panel (SRS346903, SRS346890).
(PDF)

S5 Fig Second homozygous Almond male has a similar expression profile to original homozygous Almond assayed in Fig 5. Dot plots show results of qRT-PCR assays of gene expression of genes in the Almond CNV region and pigmentation genes assayed as part of the original experiment in Fig 5. (A) Genes inside the CNV. (B) Genes outside the CNV. (C) Melanocyte-related genes. In order to complement gene expression data from the original experiment, qRT-PCR expression assays were re-run on a pair of regenerating feather bud samples from the following phenotypes: non-Almond (NA), dark Almond (DA), the original homozygous Almond for this study (HA1), and a recently obtained homozygous Almond (HA2). T-tests showed that the two homozygous Almonds were not statistically different.
(PDF)
