# Supplementary material for: A copy number variant is associated with a spectrum of pigmentation patterns in the rock pigeon (Columba livia)
Source: PLoS Genet. 2020 May 20;16(5):e1008274. doi: 10.1371/journal.pgen.1008274 (PMC7239393; doi:10.1371/journal.pgen.1008274)
Supplement: S1 Fig — This region was found to contain a CR-1-like element. Plots show normalized read-depth for resequenced pigeon genomes titled by SRA accession number. Reads were aligned to the Almond CNV region, with the gap in the genome assembly (Cliv_2.1) bridged with sequence obtained from Sanger sequencing. Representative non-Almond individuals are shown in the left column, with Almond individuals on the right. The top two rows are male, and the bottom two rows are females. The x-axis is the distance from the gap region sequence, and the y-axis shows coverage depth, normalized to the first 10000 bp of the region. All individuals have a spike in coverage in the region containing the CR-1-like transposable element sequence, with females showing a greater increase than males. The greater increase in females could be due to an abundance of CR-1-like transposable element sequence on the W chromosome. (PDF) [file pgen.1008274.s007.pdf]

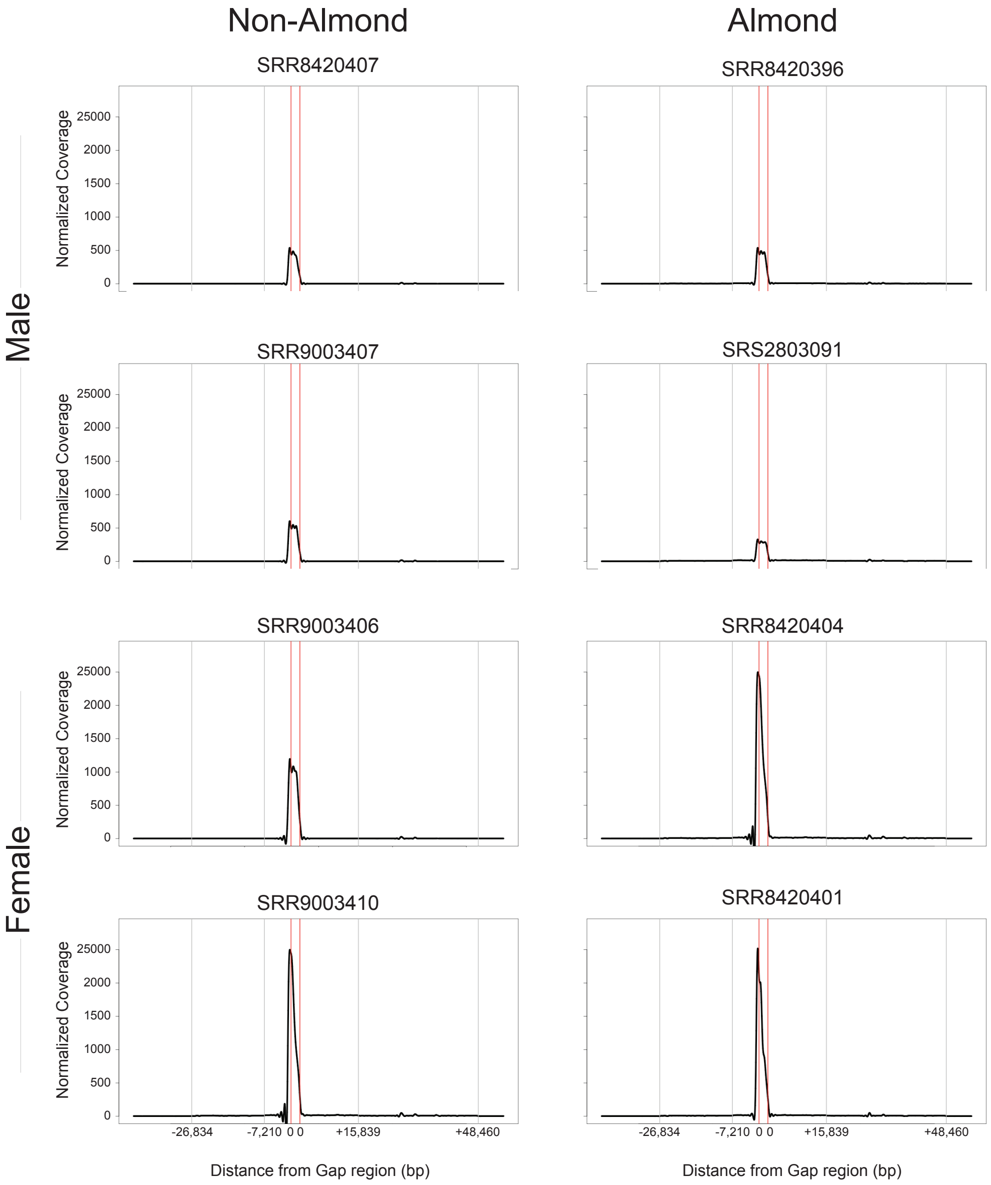

S1 Figure. Depth plots of whole-genome sequencing data around genomic gap region in the Almond CNV. This region was found to contain a CR-1-like element. Plots show normalized read-depth for resequenced pigeon genomes titled by SRA accession number. Reads were aligned to the Almond CNV region, with the gap in the genome assembly (Cliv_2.1) bridged with sequence obtained from Sanger sequencing. Representative non-Almond individuals are shown in the left column, with Almond individuals on the right. The top two rows are male, and the bottom two rows are females. The x-axis is the distance from the gap region sequence, and the y-axis shows coverage depth, normalized to the first 10000 bp of the region. All individuals have a spike in coverage in the region containing the CR-1-like transposable element sequence, with females showing a greater increase than males. The greater increase in females could be due to an abundance of CR-1-like transposable element sequence on the W chromosome.
