# Supplementary material for: A copy number variant is associated with a spectrum of pigmentation patterns in the rock pigeon (Columba livia)
Source: PLoS Genet. 2020 May 20;16(5):e1008274. doi: 10.1371/journal.pgen.1008274 (PMC7239393; doi:10.1371/journal.pgen.1008274)
Supplement: S2 Fig — We repeated the association test shown in Fig 2A, except T-check and checker birds were removed from the background (non-Almond) population. We chose this example to show selection on a modifier of Almond because we previously identified the molecular basis for this trait and knew its genomic location. The Almond-associated peak remains in the same location toward the right side of the plot. Different shades of gray indicate different genomic scaffolds (same order as Fig 2A), and the horizontal dashed grey line indicates the genome-wide significance threshold. (PDF) [file pgen.1008274.s008.pdf]

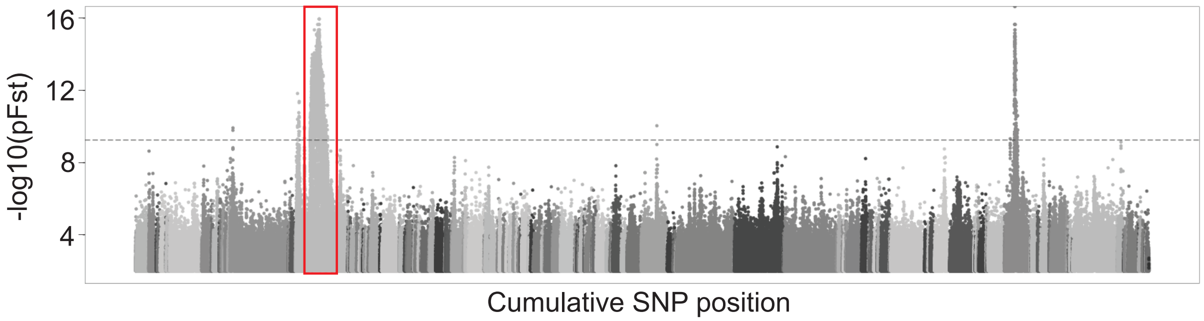

S2 Figure. Almond is associated with T-check (and/or checker) pattern alleles at the *C* locus on scaffold ScoHet_527 (red box). We repeated the association test shown in Fig. 2A, except T-check and checker birds were removed from the background (non-Almond) population. We chose this example to show selection on a modifier of Almond because we previously identified the molecular basis for this trait and knew its genomic location. The Almond-associated peak remains in the same location toward the right side of the plot. Different shades of gray indicate different genomic scaffolds (same order as Fig. 2A), and the horizontal dashed grey line indicates the genome-wide significance threshold.
