# Supplementary material for: A copy number variant is associated with a spectrum of pigmentation patterns in the rock pigeon (Columba livia)
Source: PLoS Genet. 2020 May 20;16(5):e1008274. doi: 10.1371/journal.pgen.1008274 (PMC7239393; doi:10.1371/journal.pgen.1008274)
Supplement: S3 Fig — Black dots represent results of a TaqMan copy number assay. Mean copy numbers for each phenotype are shown as red dots. These are the same data shown in Fig 4 separated by sex. (PDF) [file pgen.1008274.s009.pdf]

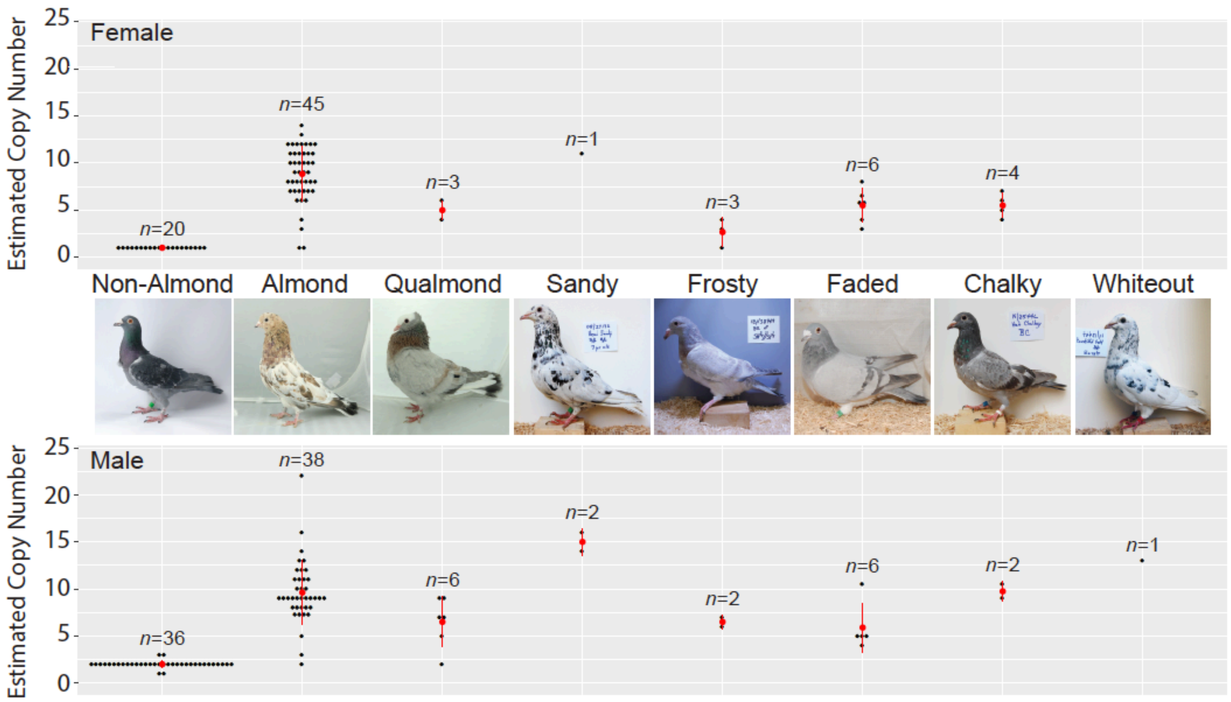

S3 Figure. *St*-linked pigmentation phenotypes show quantitative variation in the Almond CNV. Black dots represent results of a TaqMan copy number assay. Mean copy numbers for each phenotype are shown as red dots. These are the same data shown in Figure 4 separated by sex.
