# Supplementary material for: A copy number variant is associated with a spectrum of pigmentation patterns in the rock pigeon (Columba livia)
Source: PLoS Genet. 2020 May 20;16(5):e1008274. doi: 10.1371/journal.pgen.1008274 (PMC7239393; doi:10.1371/journal.pgen.1008274)
Supplement: S4 Fig — Plots in each panel compare coverage of a representative non-Almond individual (red) to mean normalized read depth for 10 female Almond birds (black) in the Almond CNV region of ScoHet5_227. SRA accession numbers for non-Almond birds are indicated above each panel. Grey dashed line at y = 1 is the coverage level expected if there is no expansion of the CNV. (A) Coverage plot for a non-Almond bird with a coverage increase in just the inner CNV region. Similar coverage increases were found in 2 out of 131 individuals analyzed in the whole genome resequencing panel (SRS346902, SRS2803087). (B) Coverage plot for a pigeon that has coverage increase of the entire CNV region but does not have the inner nested CNV duplication within the larger CNV. This configuration was found in 5 individuals in the whole genome resequencing panel (SRS346897, SRS346875, SRR8430387, SRS346889, SRS2803080). (C) Coverage plot for a non-Almond bird that has expansion of the outer CNV and further duplication of the inner CNV region. This configuration was found in two individuals from our whole genome resequencing panel (SRS346903, SRS346890). (PDF) [file pgen.1008274.s010.pdf]

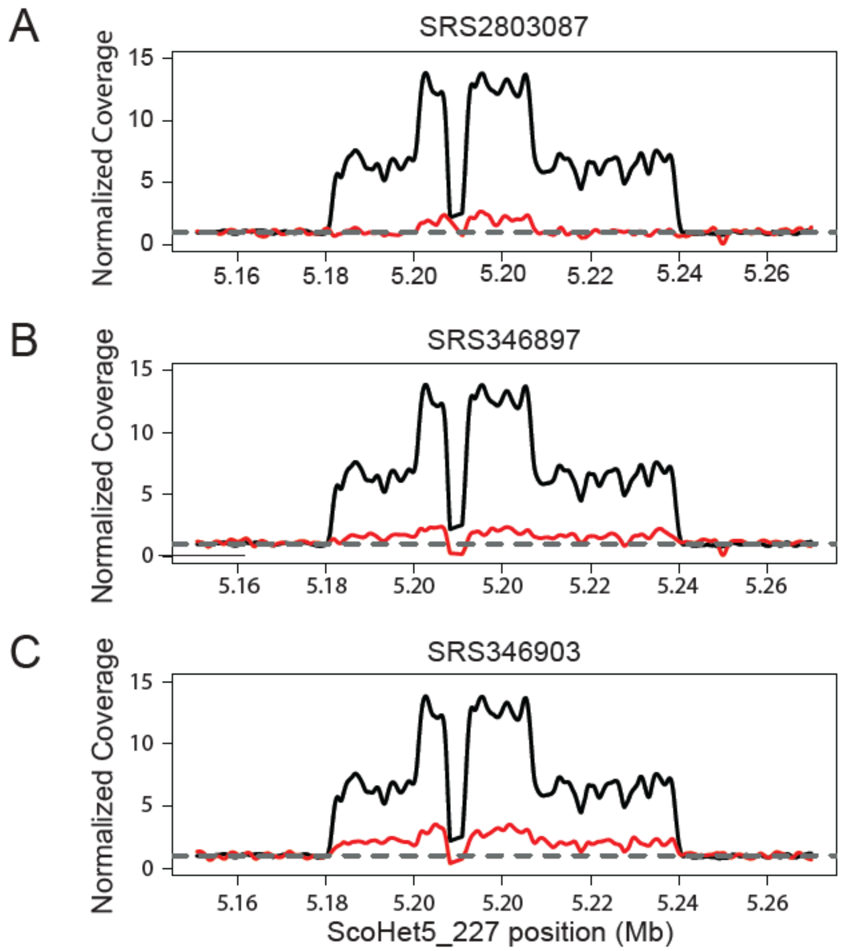

S4 Figure. Low levels of copy number increase of the Almond-associated CNV is found at low frequency in non-Almond pigeons. Plots in each panel compare coverage of a representative non-Almond individual (red) to mean normalized read depth for 10 female Almond birds (black) in the Almond CNV region of ScoHet5_227. SRA accession numbers for non-Almond birds are indicated above each panel. Grey dashed line at y=1 is the coverage level expected if there is no expansion of the CNV. (A) Coverage plot for a non-Almond bird with a coverage increase in just the inner CNV region. Similar coverage increases were found in 2 out of 131 individuals analyzed in the whole genome resequencing panel (SRS346902, SRS2803087). (B) Coverage plot for a pigeon that has coverage increase of the entire CNV region but does not have the inner nested CNV duplication within the larger CNV. This configuration was found in 5 individuals in the whole genome resequencing panel (SRS346897, SRS346875, SRR8430387, SRS346889, SRS2803080). (C) Coverage plot for a non-Almond bird that has expansion of the outer CNV and further duplication of the inner CNV region. This configuration was found in two individuals from our whole genome resequencing panel (SRS346903, SRS346890).
