# Supplementary material for: A copy number variant is associated with a spectrum of pigmentation patterns in the rock pigeon (Columba livia)
Source: PLoS Genet. 2020 May 20;16(5):e1008274. doi: 10.1371/journal.pgen.1008274 (PMC7239393; doi:10.1371/journal.pgen.1008274)
Supplement: S5 Fig — Dot plots show results of qRT-PCR assays of gene expression of genes in the Almond CNV region and pigmentation genes assayed as part of the original experiment in Fig 5. (A) Genes inside the CNV. (B) Genes outside the CNV. (C) Melanocyte-related genes. In order to complement gene expression data from the original experiment, qRT-PCR expression assays were re-run on a pair of regenerating feather bud samples from the following phenotypes: non-Almond (NA), dark Almond (DA), the original homozygous Almond for this study (HA1), and a recently obtained homozygous Almond (HA2). T-tests showed that the two homozygous Almonds were not statistically different. (PDF) [file pgen.1008274.s011.pdf]

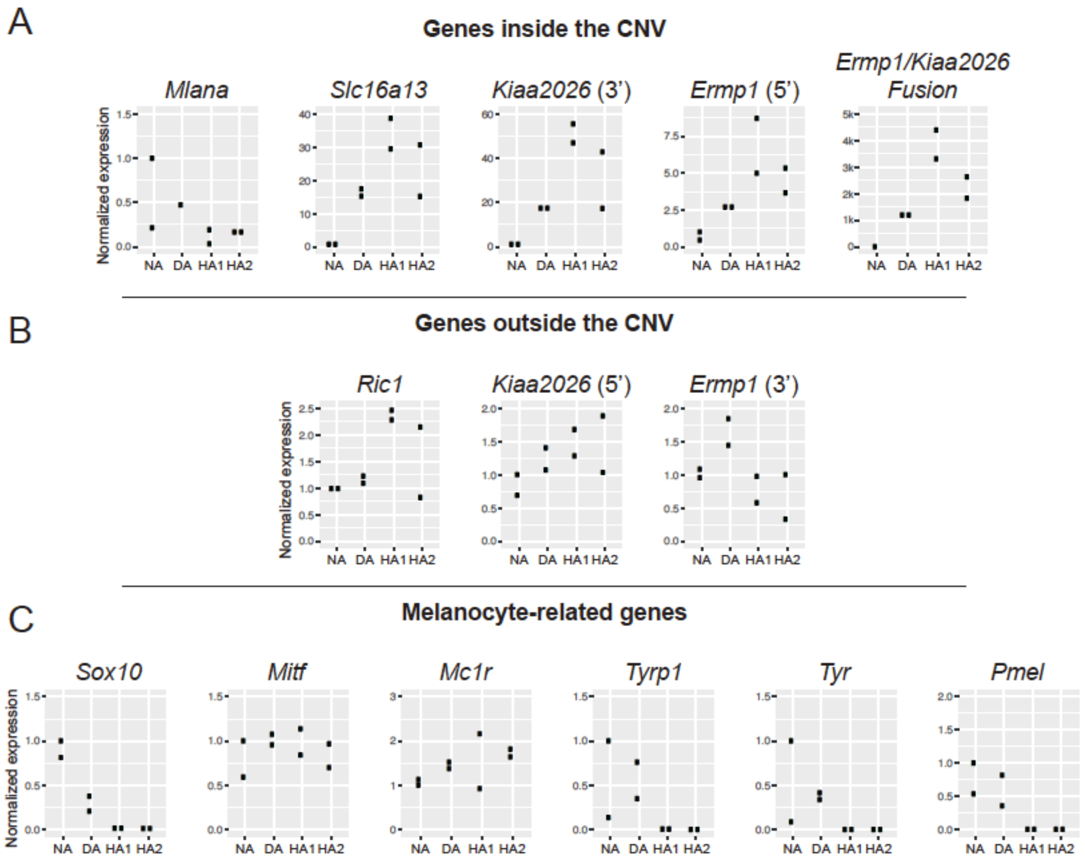

S5 Figure. Second homozygous Almond male has a similar expression profile to original homozygous Almond assayed in Figure 5. Dot plots show results of qRT-PCR assays of gene expression of genes in the Almond CNV region and pigmentation genes assayed as part of the original experiment in Figure 5. (A) Genes inside the CNV. (B) Genes outside the CNV. (C) Melanocyte-related genes. In order to complement gene expression data from the original experiment, qRT-PCR expression assays were re-run on a pair of regenerating feather bud samples from the following phenotypes: non-Almond (NA), dark Almond (DA), the original homozygous Almond for this study (HA1), and a recently obtained homozygous Almond (HA2). T-tests showed that the two homozygous Almonds were not statistically different.
